# A case of NDM-carbapenemase-producing hypervirulent *Klebsiella pneumoniae* sequence type 23 from the UK

**DOI:** 10.1099/jmmcr.0.005130

**Published:** 2018-05-17

**Authors:** Kerry J. Roulston, Tehmina Bharucha, Jane F. Turton, Katie L. Hopkins, Damien J. F. Mack

**Affiliations:** ^1^​ Department of Microbiology, Royal Free London NHS Foundation Trust, Royal Free Site, Pond Street, London NW3 2QG, UK; ^2^​ Centre for Clinical Microbiology, University College London, London, UK; ^3^​ Antimicrobial Resistance and Healthcare Associated Infections (AMRHAI) Reference Unit, National Infection Service, Public Health England, 61 Colindale Avenue, London NW9 5EQ, UK

**Keywords:** pyogenic liver abscess, hypervirulent *Klebsiella pneumoniae*, K1 capsular type, clonal complex 23, carbapenemase, metallo-β-lactamase

## Abstract

**Introduction:**

Hypervirulent capsular type K1 *Klebsiella pneumoniae* strains of clonal complex 23 (CC23) are associated with severe community-acquired pyogenic liver abscesses, often complicated by metastatic infections and significant mortality. The majority of hypervirulent strains reported are susceptible to most antibiotics except ampicillin. To the best of our knowledge, this is the first case of New Delhi metallo-β-lactamase (*bla*_NDM_)-producing hypervirulent *K. pneumoniae* from the UK.

**Case presentation:**

We present a case of pyogenic liver abscess in a 63-year-old female of Bangladeshi origin, with a recent diagnosis of pancreatic cancer. The patient was treated with piperacillin/tazobactam and blood cultures grew a fully susceptible *Escherichia coli.* Despite antimicrobial therapy and drainage of the abscess, the patient continued to deteriorate and died on day seven of admission. The fluid drained from the liver abscess grew a fully susceptible *E. coli* and a multi-drug-resistant *K. pneumoniae*. Two weeks prior to admission, a rectal screening swab grew a metallo-β-lactamase-producing *K. pneumoniae*. Molecular characterization revealed that both the *K. pneumoniae* isolates belonged to the hypervirulent K1 cluster of CC23, sequence type 23. The isolate from the rectal screen was positive for the *bla*_NDM_ metallo-β-lactamase gene.

**Conclusion:**

The emergence of carbapenemase-producing hypervirulent *K. pneumoniae* strains presents a new and significant threat to global public health. Management of these infections will be extremely challenging due to the limited treatment options available and they are likely to be associated with an even greater mortality.

## Introduction

Hypervirulent K1 *Klebsiella pneumoniae* strains are associated with severe community-acquired pyogenic liver abscesses, often complicated by metastatic infections including bacteraemia, necrotizing fasciitis, pneumonia, endopthalmitis and meningitis [1]. These infections frequently occur in younger, previously healthy adults and are associated with significant mortality [2]. This invasive syndrome emerged in the 1980s and was primarily reported in South-East Asia, but there are increasing numbers of cases being reported worldwide [1–3]. Virulence factors include capsule overproduction facilitating evasion of the immune system, and production of siderophores, which are particularly effective in sequestering iron. Some of the virulence genes are carried on a plasmid, which is also found in diverse hypervirulent strains of other capsular types, particularly K2 [2, 3].

The majority of hypervirulent *K. pneumoniae* isolates reported have been susceptible to most antibiotics except ampicillin [3, 4]. Whole-genome sequencing has been used to distinguish clonal groups of multi-drug-resistant and hypervirulent *K. pneumoniae* and although these populations are largely non-overlapping, isolates with combined virulence and resistance determinants have been detected [5]. Carbapenemase-producing *K. pneumoniae* have emerged worldwide over the last ten years [6]. These strains and their resistance plasmids have the capacity to disseminate rapidly and cause outbreaks in healthcare settings. They are frequently resistant to multiple antibiotic classes and infections can be associated with a higher mortality compared to infections with susceptible strains [7]. Hypervirulent strains of carbapenemase-producing *K. pneumoniae* have recently been reported in Argentina and China [8, 9]. To the best of our knowledge, this is the first reported case of New Delhi metallo-β-lactamase (*bla*_NDM_)-producing hypervirulent *K. pneumoniae* from the UK.

## Case report

A 63-year-old female of Bangladeshi origin presented to an Emergency Department in London, United Kingdom, in 2015 with a four day history of fever, rigors and right upper quadrant pain. She had been recently diagnosed with metastatic pancreatic cancer, and had an internal biliary stent inserted six weeks prior to admission. Two weeks prior to admission, the patient had attended hospital as a day case for a liver biopsy, at which time a rectal screening swab for carbapenemase-producing organisms was taken, which grew *K. pneumoniae*. Automated antimicrobial-sensitivity testing (AST) was performed using the Becton Dickinson Phoenix platform (BD Diagnostics) according to the European Committee on Antimicrobial Susceptibility Testing (EUCAST) guidelines. The isolate was resistant to co-amoxiclav, piperacillin/tazobactam, ceftriaxone, ceftazidime, temocillin, ertapenem, meropenem, gentamicin, amikacin, tobramycin, ciprofloxacin, levofloxacin, trimethoprim/sulfamethoxazole and fosfomycin, and susceptible to colistin, tigecycline and nitrofurantoin. Minimum inhibitory concentrations (MICs) as determined by Etest (bioMérieux) were 128 µg/ml for meropenem and 8 µg/ml for ertapenem. A positive result for the combination disc synergy test (Rosco Diagnostica) between meropenem and dipicolinic acid indicated the possible presence of a metallo-β-lactamase.

## Investigations

On admission, the patient was septic, with a temperature of 38 °C, a heart rate of 125 beats per min, blood pressure of 106/44 mmHg and a respiratory rate of 25 breaths per min. On examination, the patient had mild right upper quadrant tenderness and initial investigations revealed a white cell count of 2.8×10^10^ cells/L (2.5×10^10^ neutrophils/L), a C-reactive protein level of 318 mg/L and a normal chest X-ray. Blood cultures were taken and, after discussion with the microbiology team, the patient was started on piperacillin/tazobactam, 4.5 g intravenously, every 8 h, for presumed biliary sepsis. On day one of admission, the blood cultures grew *Escherichia coli*, which was found to be fully susceptible to all antibiotics tested including β-lactams, aminoglycosides and fluoroquinolones on day two.

**Fig. 1.**
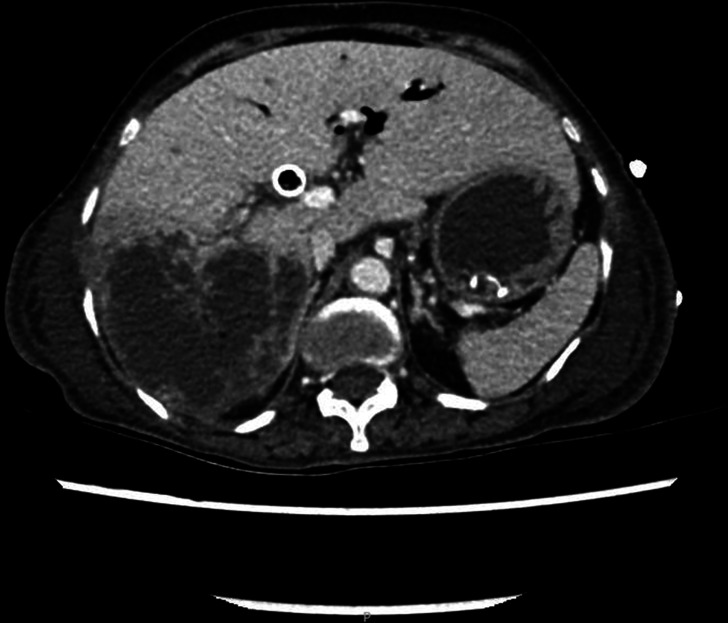
Computed tomography scan of the chest, abdomen and pelvis revealing a large (8.6×8.5×14 cm) multi-septate liver abscess within the right lobe.

On day three of admission, a computed tomography scan of the chest, abdomen and pelvis revealed a large multi-septate liver abscess within the right lobe measuring 8.6×8.5×14 cm, with a patent biliary stent and stable appearances of the large pancreatic head mass encasing the superior mesenteric vessels (Fig. 1). A pig-tail drain was inserted into the abscess under ultrasound-guidance, and heavily blood-stained fluid was drained and sent to the microbiology laboratory.

## Diagnosis

The fluid drained from the liver abscess grew *E. coli* and *K. pneumoniae*. The *E. coli* demonstrated the same susceptibility profile as the *E. coli* previously isolated from blood cultures. The *K. pneumoniae* was resistant to the same antibiotics as the *K. pneumoniae* previously isolated from the rectal screen, apart from ertapenem and meropenem, to which it was sensitive. MICs as determined by Etest (bioMérieux) were 0.047 µg/ml for meropenem and 0.047 µg/ml for ertapenem. Combination disc synergy test between cefpodoxime and cefpodoxime/clavulanic acid indicated the presence of an extended-spectrum β-lactamase.

## Treatment

On admission, the patient was treated empirically with piperacillin/tazobactam, 4.5 g intravenously, every 8 h, for presumed biliary sepsis. This was changed to co-amoxiclav, 1.2 g intravenously, every 8 h based on the AST profile of the *E. coli* isolated from blood cultures.

## Outcome and follow-up

Despite drainage and antimicrobial therapy active against the *E. coli* grown from blood cultures, the patient continued to deteriorate. The hepato-pancreato-biliary surgical team did not consider the patient to be an appropriate candidate for surgery and she died on day seven of admission. AST results for the *K. pneumoniae* isolated from the liver abscess fluid were not available until the day the patient died.

The *K. pneumoniae* isolates from the rectal screening swab and liver abscess were referred to the national reference laboratory for further molecular investigation. Molecular characterization was undertaken using nine-locus variable number tandem repeat (VNTR) analysis (at loci A, E, H, J, K, D, N1, N2 and N4) and multiplex-PCR for capsular type-specific and virulence gene targets, as described previously [10, 11]. Multiplex-PCR was used to screen for KPC, OXA-48-like, NDM and VIM acquired carbapenemase genes, as described previously [12]. Next-generation sequencing (NGS) was also performed by the Genomic Services and Development Unit (National Infection Service, Public Health England, London, UK) on an Illumina Hi-Seq platform following extraction on a QIASymphony instrument.

Both isolates were confirmed as capsular type K1, and VNTR and NGS identified both strains as belonging to the K1 cluster of clonal complex 23 (CC23) (VNTR profile 3,5,2,7,0,1,4,4,1), both of classical sequence type 23 (ST23). Both isolates were positive for *wcaG*, which encodes capsular fucose synthesis and is always associated with K1 isolates, and negative for *rmpA*, a positive regulator of extracapsular polysaccharide synthesis. The isolate from the rectal screening swab was positive for the *bla*_NDM_ metallo-β-lactamase gene (confirmed as *bla*_NDM-1_ by NGS), but the isolate from the liver abscess was negative for all acquired carbapenemase genes tested, in keeping with the phenotypic antibiotic-susceptibility results. NGS revealed the presence of virulence determinants typically associated with hypervirulent K1 *K. pneumoniae* strains of CC23 in both isolates. These included: *allS*, which is associated with allantoin metabolism, various iron acquisition systems, aerobactin (*iucABCD-iutA*), the ferric uptake operon *kfu*ABC, yersiniabactin (*ybt*AEUPQRSTX), colibactin (*clb*ABCDEFGHIJKLMNOPQR), salmochelin (*iroBCDN*), a tellurite resistance operon (*ter*ABCDEWXZ) and *rmpA2* (a variant of *rmpA*).

## Discussion

As far as we are aware, this is the first reported case of carbapenemase-producing ST23 *K. pneumoniae* in the UK. Although reports of carbapenemase-producing hypervirulent *K. pneumoniae* strains have so far been scarce, the emergence of such strains is particularly concerning due to the combination of hypervirulence and multi-drug resistance. Management of carbapenemase-producing hypervirulent *K. pneumoniae* infections will be extremely challenging due to the limited treatment options available and these strains are likely to be associated with an even greater mortality. Rapid diagnosis and appropriate management has the potential to improve outcomes and minimize the risk of metastatic complications [1]. The prospect of potentially untreatable invasive infection in young, previously healthy adults presents a new and significant threat to global public health.
